# NF-κB/IL-6 axis drives impaired corneal wound healing in aqueous-deficient dry eye

**DOI:** 10.3389/fimmu.2025.1684290

**Published:** 2025-10-24

**Authors:** Cuipei Lin, Jiaxin Wu, Yuxin Jing, Jingbin Xie, Jiayan Xiang, Qiwei Fan, Jiangman Liu, Jingheng Du, Xiukui Tan, Zhudan Zhuang, Yunxia Xue, Ting Fu, Jun Liu, Zhijie Li

**Affiliations:** ^1^ International Ocular Surface Research Center, Institute of Ophthalmology, and Key Laboratory for Regenerative Medicine, Jinan University, Guangzhou, China; ^2^ Department of Ophthalmology, The First Affiliated Hospital of Jinan University, Guangzhou, China

**Keywords:** dry eye disease, corneal wound healing, inflammation mechanisms, NF‐κB, IL‐6, re‐epithelialization, nerve regeneration

## Abstract

Dry eye disease (DED) commonly leads to compromised corneal epithelial integrity and chronic ocular surface inflammation; however, the precise molecular mechanisms underlying impaired corneal healing remain incompletely understood. In this study, we established an aqueous‐deficient DED mouse model via extraorbital lacrimal gland excision (LGE) to investigate the cellular and molecular events contributing to delayed corneal repair following epithelial injury. Our results demonstrated significant delays in corneal re‐epithelialization and nerve regeneration in aqueous‐deficient mice, accompanied by increased infiltration of neutrophils and γδ T cells. Transcriptomic profiling revealed robust activation of NF‐κB‐dependent inflammatory signaling and IL‐6 pathways, alongside marked suppression of genes associated with essential metabolic processes involved in tissue repair. Targeted pharmacological inhibition of NF‐κB using caffeic acid phenethyl ester (CAPE), or neutralization of IL‐6 via topical anti-IL‐6 antibody administration, effectively attenuated immune cell infiltration, accelerated epithelial regeneration, and enhanced nerve recovery. Collectively, our findings suggest that NF-κB/IL-6 signaling plays a key role in sustaining inflammation that impairs corneal healing, and that targeting this axis partially improves epithelial and neural outcomes.

## Highlights

- LGE-induced aqueous deficiency delays re-epithelialization and impairs corneal sensory nerve regeneration after epithelial injury.
- Persistent accumulation of Ly6G^+^ neutrophils and γδ T cells sustains an inflammatory milieu that disrupts repair.
- NF‐κB/IL‐6 signaling is implicated as a contributory inflammatory pathway that impairs epithelial and nerve regeneration.
- Blocking NF-κB or neutralizing IL-6 reduces immune-cell infiltration, accelerates epithelial closure, and enhances nerve recovery.

## Introduction

Dry eye disease (DED) is a multifactorial ocular disorder characterized primarily by tear film instability, resulting from inadequate tear secretion, excessive evaporation, or altered tear film composition (1), significantly impacting visual function and quality of life worldwide (2). Clinically, DED is categorized into aqueous-deficient, evaporative, and mixed types (3). Beyond lubrication, the tear film delivers critical growth factors and anti-inflammatory mediators crucial for maintaining epithelial homeostasis, modulating inflammation, and facilitating ocular surface repair (4). Consequently, tear film dysfunction in DED can lead to persistent epithelial defects, chronic inflammation, and increased risk of vision loss.

Due to its exposed anatomical location, the cornea is particularly vulnerable to environmental injuries. Corneal epithelial damage accounts for nearly half of ophthalmic emergencies (5, 6), and the rising prevalence of corneal refractive surgery further elevates the incidence of corneal trauma. Simultaneously, the increasing prevalence of DED exacerbates injury susceptibility within compromised ocular environments, emphasizing the urgency to understand the precise mechanisms through which DED disrupts corneal healing.

Corneal wound repair is an intricately regulated process comprising inflammation initiation, epithelial proliferation, re‐epithelialization, nerve regeneration, and stromal remodeling (7). Effective corneal repair critically depends on a balanced immune response. Neutrophils rapidly migrate to injury sites, removing debris through enzymatic action and phagocytosis (8). γδ T cells regulate epithelial regeneration by secreting cytokines such as IL‐17A and IL‐22, influencing neutrophil recruitment and epithelial cell proliferation (9). Additionally, NK cells (10), mast cells (11), distinct macrophage subsets (CCR2^─^ and CCR2^+^) (12), and type‐2 innate lymphoid cells (13) coordinate inflammation resolution, directly influencing healing outcomes. Imbalances within this immune network, particularly in inflammatory ocular diseases like DED, significantly disrupt corneal repair.

Although recent advances have elucidated aspects of DED pathology, the impact on epithelial and neural regeneration during corneal healing, including immune response dynamics, remains poorly defined. Here, we hypothesized that DED exacerbates corneal healing impairment by intensifying inflammation. Using an aqueous-deficient DED mouse model established by extraorbital lacrimal gland excision (LGE) (14), we examined epithelial repair, nerve regeneration, and immune cell infiltration following corneal injury. Transcriptomic profiling identified significant upregulation of NF‐κB and IL‐6 signaling pathways as central mechanisms driving excessive inflammation. Finally, we explored whether pharmacological blockade of NF‐κB or IL‐6 pathways could alleviate delayed wound healing and nerve regeneration defects under dry eye conditions.

## Materials and methods

### Animals

Male C57BL/6 mice (7–8 weeks old), verified to be free of ocular surface abnormalities, were consistently obtained from the Medical Experimental Animal Center (Guangdong, China). Animals were uniformly housed under strictly standardized laboratory conditions, including a controlled 12-hour light/dark cycle, stable ambient temperature (22 ± 2°C), and consistent humidity levels (50–60%), with unrestricted access to standardized food and water throughout the study period.

All animal experiments were rigorously performed in strict accordance with the ARVO Statement for the Use of Animals in Ophthalmic and Vision Research. Experimental protocols were reviewed, approved, and regularly monitored by the Institutional Animal Care and Use Committee (IACUC) of Jinan University (Approval No.: 20200318-22), ensuring full compliance with internationally recognized animal welfare standards. At predetermined experimental endpoints, animals were consistently euthanized using standardized protocols involving CO_2_ overdose followed by cervical dislocation, ensuring ethical and humane termination according to established guidelines.

### Extraorbital lacrimal gland excision

A standardized unilateral extraorbital lacrimal gland excision (LGE) was performed to consistently induce aqueous-deficient dry eye conditions, strictly adhering to previously validated methods (14–17). Briefly, mice were anesthetized via intraperitoneal injection of sodium phenobarbital (25–50 mg/kg) under uniform and controlled conditions. A precisely measured skin incision (~1 cm) was systematically made midway between the lateral canthus and the ear. Using standardized surgical instruments, underlying tissues were meticulously dissected to reliably expose and completely remove the extraorbital lacrimal gland. Hemostasis was consistently verified following gland removal, and the surgical incision was uniformly closed using gentle skin sutures to promote reproducible healing outcomes.

Sham-operated control mice underwent an identical surgical procedure, including anesthesia, incision, and tissue manipulation, except without gland removal, ensuring strict control conditions for all comparative analyses.

### Aqueous tear secretion measurement

Basal tear secretion was assessed using a modified Schirmer’s test. Schirmer tear test strips (Guangxia Medical Instruments, Suzhou, China) were precisely cut to 2.5 mm width. Each strip was placed gently into the lateral lower conjunctival fornix without anesthesia and left in place for exactly 5 minutes. The wetted strip length (in millimeters) was recorded immediately after removal. This procedure was conducted three times daily for each eye, and the average of these measurements was taken as the representative tear secretion level. All tests were performed between 9:00 and 11:00 AM under controlled environmental conditions (22 ± 2°C, 50–60% humidity) to minimize diurnal variation and ensure reproducibility.

### Corneal fluorescein staining

Corneal epithelial integrity and damage were systematically assessed using standardized sodium fluorescein staining protocols as previously validated (18, 19). Briefly, a precise volume (5 μL) of 2% sodium fluorescein solution was consistently instilled into the conjunctival sac of each mouse eye. Corneal staining patterns were then uniformly examined under a stereomicroscope equipped with a standardized cobalt blue filter to ensure reproducible visualization conditions.

For objective quantification, the corneal surface was consistently divided into five standardized regions: central, nasal, temporal, superior, and inferior. Each region was scored independently using a validated and standardized intensity scale ranging from 0 to 3, where 0 represented no visible staining and 3 indicated severe epithelial damage (20). The total fluorescein staining score per eye was systematically calculated as the sum of the scores across all five regions, yielding a maximum possible score of 15, providing a reproducible quantitative measure of overall epithelial damage.

All scoring was performed by the same trained examiner blinded to the treatment groups, ensuring objectivity and reproducibility of the evaluation.

### Corneal wound healing model

Two weeks post-establishment of the aqueous-deficient dry eye condition, corneal epithelial abrasions were induced in mice following standardized procedures previously reported (21–24). Briefly, mice received an intraperitoneal injection of sodium phenobarbital (25–50 mg/kg) for anesthesia. The central corneal region (2-mm diameter) was precisely demarcated with a trephine. Using a dissecting microscope for enhanced precision, epithelial layers within the marked area were carefully removed with a golf club-shaped corneal scraper (Accutome, Malvern, PA), without disturbing the underlying stromal tissue. Wound recovery was monitored at predetermined intervals using 2% sodium fluorescein staining, and the area of the residual epithelial defect was systematically recorded via imaging for quantitative assessment.

### Histological analysis

Histological analyses were performed using standardized, reproducible methods. Briefly, lacrimal glands and intact eyeballs, including the cornea and conjunctiva, were carefully harvested and consistently fixed overnight at 4°C in freshly prepared 4% paraformaldehyde. Tissues underwent standardized dehydration through a graded ethanol series, were embedded uniformly in paraffin, and sectioned at precisely 5 μm thickness using a calibrated microtome.

Histological sections were systematically stained with hematoxylin and eosin (H&E) reagent, strictly following standardized and established staining protocols.

For conjunctival analyses, goblet cell quantification was performed consistently under a 20× objective lens across the entire microscopic field to ensure reproducibility and accuracy. For corneal epithelial thickness measurements, central corneal sections were uniformly imaged under identical conditions, and epithelial thickness was precisely quantified using a calibrated linear measurement tool provided by the SoftWorx image analysis software (Applied Precision, Issaquah, WA, USA).

All measurements and evaluations were systematically conducted in a blinded manner to minimize observer bias and ensure reproducible outcomes.

### Immunostaining

Immunostaining was performed using standardized and reproducible procedures. Briefly, mouse eyeballs were uniformly fixed in 4% paraformaldehyde for exactly 1 hour at room temperature. Under a dissecting microscope, intact corneas including the limbal region were carefully dissected using standardized fine corneal scissors. The isolated corneal tissues were then consistently blocked with 2% bovine serum albumin (BSA) for 15 minutes to prevent nonspecific antibody binding, followed by permeabilization with 0.2% Triton X‐100 in 2% BSA solution for an additional standardized duration of 15 minutes.

Subsequently, whole corneas were systematically incubated overnight at 4°C with one of the following standardized primary antibodies: i) Anti‐mouse Ly6G conjugated with fluorescein isothiocyanate (FITC; 1:100 dilution; BD Biosciences, Cat. No. 551460); ii) Anti‐mouse GL3 conjugated with phycoerythrin (PE; 1:100 dilution; BD Biosciences, Cat. No. 553178); iii) Anti‐β‐III tubulin conjugated with NL557 (1:10 dilution; R&D Systems, Cat. No. NL1195R).

After the primary antibody incubation, corneas underwent standardized washing with phosphate-buffered saline (PBS), three times for precisely 5 minutes each (total 15 minutes), to remove unbound antibodies. Corneas were then radially incised and flattened uniformly onto glass slides. Each sample was mounted consistently with antifade mounting medium containing 1 μM DAPI (Sigma‐Aldrich, Cat. No. 28718‐90‐3).

Immunofluorescence imaging was systematically performed and analyzed using a standardized DeltaVision Image System (Applied Precision, Issaquah, WA, USA), ensuring uniformity and reproducibility across all experimental groups. Acquisition parameters were identical among groups; scale bars are indicated in all panels.

### Corneal wound healing assessment

Corneal wound healing was systematically evaluated through three standardized parameters: epithelial cell proliferation, immune cell infiltration, and nerve fiber regeneration.

**Epithelial Cell Proliferation**: To accurately assess epithelial proliferative activity, the cornea was consistently divided into five concentric zones (zones 1–5) radiating from the limbal region to the central cornea, with zones 3–5 specifically corresponding to the abraded area (**Supplementary Figure 2**). Mitotic basal epithelial cells were visualized by standardized DAPI staining protocols and quantified consistently under a 40× objective lens across 17 predefined microscopic fields spanning the entire corneal diameter. The total mitotic cell counts from these standardized fields provided a reproducible quantitative index of epithelial proliferation.

**Immune Cell Infiltration**: Inflammatory cell recruitment was quantified by standardized whole-mount immunofluorescence staining methods, focusing specifically on neutrophils and γδ T cells. For each cornea, immune cells were systematically counted in four representative microscopic fields under a 40× objective lens, as precisely defined in **Supplementary Figure 2**. The mean number of positively stained immune cells per field served as a reliable and reproducible measure of immune infiltration into the injured cornea.

**Nerve Fiber Regeneration**: Corneal nerve regeneration was rigorously assessed by standardized quantification of nerve density using Imaris 6.2 software (Bitplane AG, Zurich, Switzerland). Immunofluorescent labeled β‐III tubulin‐positive nerve fibers within a centrally defined 1500 μm × 1500 μm region of each cornea were imaged and analyzed. Total nerve fiber length was measured systematically, and nerve density was precisely expressed as total fiber length per unit area (mm/mm²).

This standardized, multiparametric evaluation provided a robust and highly reproducible methodological framework to comprehensively assess corneal wound healing dynamics, encompassing epithelial, immunological, and neural components across all experimental conditions.

### Corneal sensitivity measurement

Corneal mechanical sensitivity was consistently assessed using a standardized protocol with a handheld Cochet-Bonnet esthesiometer (Luneau SAS, France), according to previously validated methods (25–27). The esthesiometer utilizes a calibrated, retractable nylon monofilament with adjustable lengths from 0.5 to 6.0 cm, allowing precise delivery of tactile stimuli. During each measurement, the central corneal region was carefully touched perpendicularly with the standardized 0.5‐cm monofilament. The stimulus was uniformly applied four times per cornea, and a positive blink reflex response to at least one stimulus was consistently recorded as indicating preserved corneal sensitivity.

To ensure rigorous reproducibility and eliminate potential bias, all measurements were systematically conducted under double-blind conditions by a single, trained examiner throughout the experimental procedure.

### RNA‐seq

RNA sequencing (RNA‐Seq) and subsequent bioinformatic analyses were performed rigorously according to previously published and validated methods (28, 29). Specifically, mice were euthanized exactly 18 hours post-corneal epithelial abrasion, after which injured corneal tissues were carefully dissected and immediately snap-frozen in liquid nitrogen to preserve RNA integrity. Total RNA extraction was consistently carried out using TRIzol reagent (Invitrogen, Carlsbad, CA, USA), strictly adhering to the manufacturer’s recommended protocol. RNA purity and concentration were uniformly quantified using a standardized NanoDrop spectrophotometer (Thermo Fisher Scientific, Waltham, MA, USA).

Library preparation and sequencing were systematically conducted by the Beijing Genome Institute (BGI, Shenzhen, China), ensuring consistency across samples. 100 bp paired-end sequencing were performed on the BGISEQ-500 platform. At least 20 million raw reads per sample were generated. Across samples, uniquely mapped reads averaged 69.55% ± 1.31%, and rRNA content was < 2.5%. Differential expression analysis was conducted with the DESeq2 package (v1.38.3) in R. Genes with an adjusted P-value (FDR) < 0.05 and absolute log2 fold change > 1 were considered significantly differentially expressed. For downstream bioinformatic analysis, standardized gene ontology (GO) enrichment analysis was performed using the Gene Ontology Resource (http://geneontology.org/). Network analyses of significantly differentially expressed genes were uniformly conducted using Cytoscape software (version 3.10.2) to ensure reproducible identification of key signaling pathways and molecular interactions.

Each sequencing experiment included multiple biological replicates to ensure robustness, reproducibility, and accuracy of the data analysis.

### Quantitative real‐time PCR

Total RNA was consistently extracted from harvested corneal tissues using the RNA Simple Total RNA Kit (Tiangen Biotech, Beijing, China; Cat. No. DP419) according to the manufacturer’s standardized protocol. Complementary DNA (cDNA) synthesis was rigorously performed from purified RNA samples using the ReverTra Ace qPCR RT Kit (Toyobo Co., Osaka, Japan; Cat. No. FSQ‐101), precisely following the provided instructions.

Quantitative real‐time PCR (qPCR) was conducted on a standardized real‐time PCR detection system utilizing the THUNDERBIRD SYBR qPCR Mix (Toyobo; Cat. No. QPS‐201), ensuring uniformity across all experimental runs. Gene‐specific primers, carefully designed and validated for accuracy and efficiency, were employed to quantify the expression levels of targeted genes. Detailed primer sequences used throughout this study are presented comprehensively in **Table 1**.

**Table 1:** PCR primers used in this study.

Gene name	Primer sequence	
*Nfkb1*	Forward	5′-GGAAGACAAGGAGCAGGACAT-3′
	Reverse	5′-AGCGTGGAGGTGGATGATG-3′
*Il6*	Forward	5′-GACTTCCATCCAGTTGCCTT-3′
	Reverse	5′-TGTGTAATTAAGCCTCCGACT-3′
*Ki67*	Forward	5′-CAATCCAACTCAAGTAAACGG-3′
	Reverse	5′-TTTGGTATCTTGACCTTCCC-3′
*Pcna*	Forward	5′-TCCTTGGTACAGCTTACTCT-3′
	Reverse	5′-CGGCATCTTTATTACACAGC-3′
*Gapdh*	Forward	5′-CAAGGACACTGAGCAAGAG-3′
	Reverse	5′-TGCAGCGAACTTTATTGATG-3′

All qPCR reactions were independently repeated in triplicate to guarantee experimental reliability and reproducibility.

### Pharmacological agent administration

To inhibit IL‐6 signaling, anti‐mouse IL‐6 monoclonal antibody (R&D Systems, Cat. No. MAB406) was consistently applied topically to the ocular surface of mice at a standardized dosage of 5 μL (1 mg/mL diluted in phosphate‐buffered saline, PBS). Treatment commenced immediately following corneal epithelial abrasion and was repeated at precise 6‐hour intervals, following previously established protocols (30). Control groups received an equivalent dosage of an isotype‐matched IgG antibody (R&D Systems, Cat. No. MAB005) under identical conditions and schedules.

To suppress NF‐κB activation, mice were treated intraperitoneally with caffeic acid phenethyl ester (CAPE; Selleck Chemicals, Cat. No. S7414), administered at a precisely calculated dosage of 10 μmol/kg body weight every other day, according to standardized and previously validated methods (31–33). Vehicle control animals received intraperitoneal injections of an equal volume of PBS, administered at the same intervals and conditions as the CAPE‐treated group.

All treatments were performed consistently under standardized experimental conditions to ensure reproducibility and reliability of the data.

### Randomization and blinding

Mice were randomly assigned to experimental groups using a computer-generated randomization sequence. All treatments, surgical procedures, outcome assessments (wound area measurement, cell counting, and esthesiometry), and data analyses were conducted by investigators blinded to group allocation. Drug solutions and vehicle controls were prepared by an independent researcher and coded to maintain blinding throughout the study.

### Statistical analysis

All statistical analyses were conducted using SPSS statistical software (version 16.0; IBM Corp., Armonk, NY, USA). Data obtained from multiple independent experiments are expressed as mean ± standard deviation (SD). Comparisons among three or more experimental groups were systematically performed using one-way analysis of variance (ANOVA), followed by appropriate *post hoc* analyses (e.g., Tukey’s or Bonferroni tests) to precisely determine specific group differences. All data were tested for normality (Shapiro-Wilk test) and homogeneity of variances (Levene’s test). Data that did not meet ANOVA assumptions were analyzed using the Kruskal-Wallis test followed by Dunn’s *post-hoc* test. Statistical significance was consistently set at P < 0.05. Each experiment was repeated at least three times under identical conditions to ensure reproducibility and reliability of the results.

## Results

### Extraorbital lacrimal gland excision induces stable aqueous-deficient dry eye phenotype and ocular surface alterations

To reproducibly establish a stable aqueous‐deficient dry eye disease (DED) model, extraorbital lacrimal gland excision (LGE) was systematically performed in mice following previously validated protocols (14–17, 34, 35). Age‐matched control animals underwent standardized sham surgeries without gland removal. Gross examination consistently confirmed successful exposure and complete excision of the lacrimal gland during surgery (**Supplementary Figures 1A, B**). Histological evaluation of excised tissues by hematoxylin and eosin staining uniformly revealed typical glandular morphology, characterized by intact acinar structures (**Supplementary Figure 1C**).

Two weeks following surgery, LGE‐treated mice exhibited significantly reduced tear secretion compared to sham‐operated controls, as reliably quantified by standardized Schirmer tear test strip (**Supplementary Figure 1D**). Histological analyses of conjunctival tissues further demonstrated a reproducible and significant reduction in goblet cell density in LGE‐treated animals (**Supplementary Figure 1E**). Standardized corneal fluorescein staining and histological analyses of central cornea consistently revealed significantly elevated epithelial staining scores at both one and two weeks post‐LGE, indicative of persistent epithelial surface disruption (**Supplementary Figures 1F, G**).

Additionally, immunofluorescent staining of corneal nerves using the standardized β‐III tubulin marker clearly demonstrated a progressive and significant defect in corneal nerve fibers in LGE‐treated mice compared to controls, particularly notable at two weeks post‐surgery (**Supplementary Figure 1H**). However, there is no difference in corneal sensitivity between control and LGE-treated mice (**Supplementary Figure 1I**).

Collectively, these rigorously obtained and reproducible findings confirm that extraorbital LGE reliably induces a stable aqueous‐deficient DED phenotype, characterized by markedly reduced tear secretion and well─defined pathological changes in both conjunctival and corneal tissues.

### LGE impairs corneal re-epithelialization following epithelial abrasion

**Figure 1:**
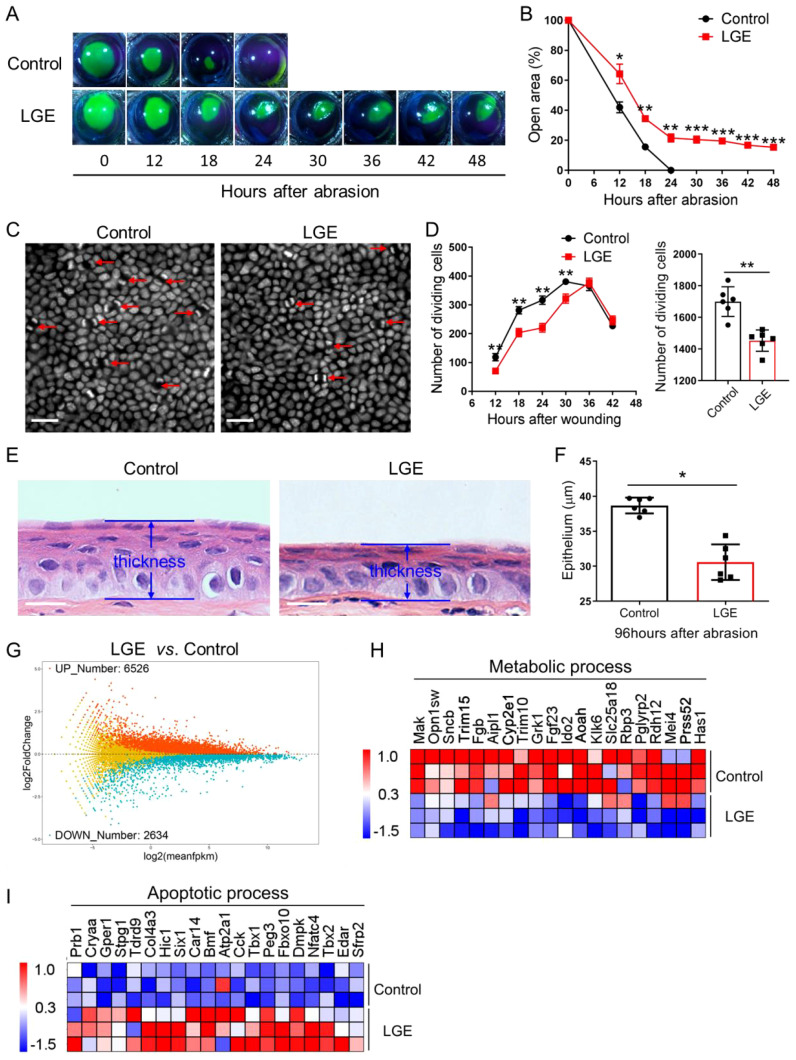
Delayed corneal epithelial wound healing in LGE-induced dry eye model. **(A)** Representative fluorescein sodium staining images showing the wounded corneal area at indicated time points (0–48 hours) following central epithelial abrasion in control and LGE‐treated mice. **(B)** Quantification of residual corneal wound area over time reveals a significantly slower healing rate in LGE‐treated mice compared to controls. **(C)** DAPI‐stained whole‐mount images of corneal epithelium from control and LGE‐treated mice at 24 hours post‐injury. Red arrows indicate dividing epithelial cells characterized by coupled nuclei (scale bars = 25 μm). **(D)** Left panel: time‐course analysis of dividing basal epithelial cells in control and LGE‐treated mice from 12 to 42 hours after abrasion. Right panel: cumulative number of dividing cells across all time points. Cell counts were conducted across 17 fields per cornea (as illustrated in **Supplementary Figure 2**). **(E)** Hematoxylin and eosin (H&E) staining of paraffin‐embedded corneal sections at 96 hours after abrasion. Images illustrate reduced epithelial thickness in LGE‐treated mice. **(F)** Quantitative comparison of central corneal epithelial thickness between groups at 96 hours post‐injury. **(G)** An MA plot showing the effect of LGE treatment on the expression of corneal genes at 18 hours after epithelial abrasion. **(H, I)** Heatmaps depict the changes in expression of corneal genes involved in metabolic **(H)** and apoptotic **(I)** process in LGE‐treated mice compared to control mice (top 20 differentially expressed genes are shown). All data are presented as mean ± SD. Sample size: *n* = 6 mice per group **(B, D, F)**; *n* = 3 independent experiments (4 mice per experiment in each group; **(G–I)**). Statistical significance: *P < 0.05, **P < 0.01, and ***P < 0.001.

To systematically evaluate the impact of aqueous‐deficient dry eye conditions on corneal wound healing, central epithelial abrasions were consistently induced in both LGE‐treated and sham‐operated control mice. Corneal re‐epithelialization was precisely monitored over a standardized 48‐hour observation period. Sodium fluorescein staining revealed reproducibly delayed wound closure in the LGE‐treated group compared to controls, with significant differences clearly observed from 12 to 48 hours post‐injury (**Figures 1A, B**). Quantitative analysis confirmed that the residual epithelial defect area remained consistently and significantly larger in LGE‐treated mice throughout the assessment period.

To further rigorously assess epithelial regenerative capacity, mitotic basal epithelial cells were visualized using standardized DAPI staining. Quantitative analysis demonstrated consistently fewer dividing cells in LGE‐treated mice relative to control groups, as clearly illustrated by representative images and quantified in the detailed time‐course analysis (**Figures 1C, D**). Moreover, qPCR analysis showed that proliferation marker genes, *Ki67* and *Pcna* were both decreased in LGE-treated mice compared to control mice (**Supplementary Figure 4**). Cumulative quantification across the entire observation window confirmed a significant reduction in total epithelial proliferative activity in LGE‐treated mice compared to controls.

Histological evaluations at 96 hours post‐injury reproducibly revealed significantly thinner corneal epithelial layers in LGE‐treated mice relative to control animals (**Figure 1E**). Morphometric analysis of these standardized histological sections further confirmed a statistically significant decrease in epithelial thickness in the LGE‐treated group (**Figure 1F**).

Furthermore, transcriptomic profiling conducted under rigorously standardized conditions demonstrated substantial gene expression alterations in LGE‐treated corneas. Specifically, RNA‐seq analysis identified 6526 significantly upregulated and 2634 significantly downregulated genes in the LGE group compared to controls (**Figure 1G**). Gene ontology analysis further highlighted a consistent downregulation of metabolic‐process‐related genes and significant upregulation of apoptosis‐associated genes in the LGE‐treated corneas (**Figures 1H, I**).

Taken together, these reproducibly obtained results clearly demonstrate that LGE‐induced aqueous‐deficient dry eye consistently and significantly delays corneal re‐epithelialization. This impairment is characterized by notably slower wound closure, markedly reduced epithelial proliferation, attenuated regeneration capacity, and significant transcriptomic alterations indicative of impaired metabolic processes and enhanced apoptosis following corneal injury.

### LGE impairs corneal nerve regeneration and delays sensory recovery after epithelial abrasion

**Figure 2:**
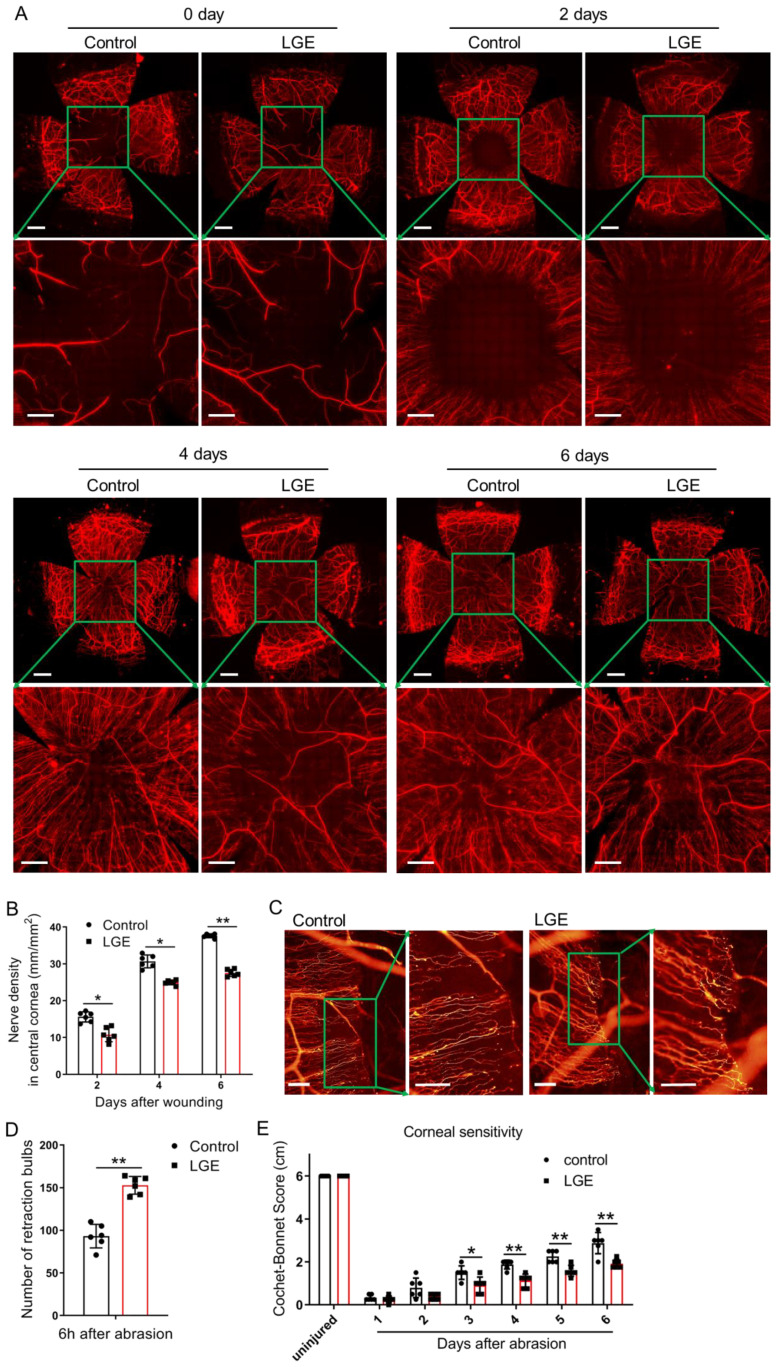
LGE impairs corneal nerve regeneration and sensory recovery following epithelial abrasion. **(A)** Representative immunofluorescence images of corneal nerve fibers at 0, 2, 4, and 6 days after epithelial abrasion in control and LGE‐treated mice. Corneal nerves were visualized using an anti‐β‐III tubulin antibody conjugated with NL557. Upper panels show wide‐field views (scale bars = 500 μm); lower panels show magnified views of the central cornea (scale bars = 200 μm). **(B)** Quantification of corneal nerve density in the central 1500 μm × 1500 μm region using Imaris 6.2 software (as illustrated in **Supplementary Figure 3**). **(C)** Representative images of retraction bulbs in the central cornea at 6 hours post‐abrasion (scale bars = 40 μm). **(D)** Quantification of retraction bulb numbers in control and LGE‐treated mice. **(E)** Corneal mechanical sensitivity measured using a Cochet‐Bonnet esthesiometer at days 1–6 post‐abrasion. LGE‐treated mice exhibited reduced corneal sensitivity compared to controls at days 3–6. All data are presented as mean ± SD. Sample size: *n* = 6 mice per group **(B, D, E)**. Statistical significance: *P < 0.05, and **P < 0.01.

To rigorously assess the effects of aqueous‐deficient dry eye on corneal nerve regeneration, standardized whole‐mount immunostaining for β‐III tubulin was performed systematically at 2, 4, and 6 days following corneal epithelial abrasion. Quantitative analysis consistently revealed a significant reduction in central corneal nerve fiber density in LGE‐treated mice compared with controls across all evaluated time points (**Figures 2A, B**).

To precisely investigate early‐stage nerve degeneration, retraction bulbs—defined morphological indicators of axonal injury—were systematically quantified at a standardized early time‐point (6 hours post‐abrasion). Results consistently showed a significantly greater number of retraction bulbs in LGE‐treated corneas relative to controls, indicating increased axonal disruption in the early phase following injury (**Figures 2C, D**).

The functional recovery of corneal sensory nerves was accurately evaluated using standardized measurements of mechanical sensitivity obtained via a Cochet‐Bonnet esthesiometer. LGE‐treated mice reproducibly displayed significantly reduced corneal sensitivity compared to controls at days 3–6 post‐abrasion (**Figure 2E**), clearly indicating delayed sensory recovery.

Collectively, these standardized and reproducible results strongly demonstrate that aqueous‐deficient dry eye conditions induced by LGE substantially impair both structural regeneration and functional sensory restoration of corneal nerves after epithelial injury.

### LGE enhances inflammatory cell infiltration and upregulates immune-related gene expression following corneal abrasion

**Figure 3:**
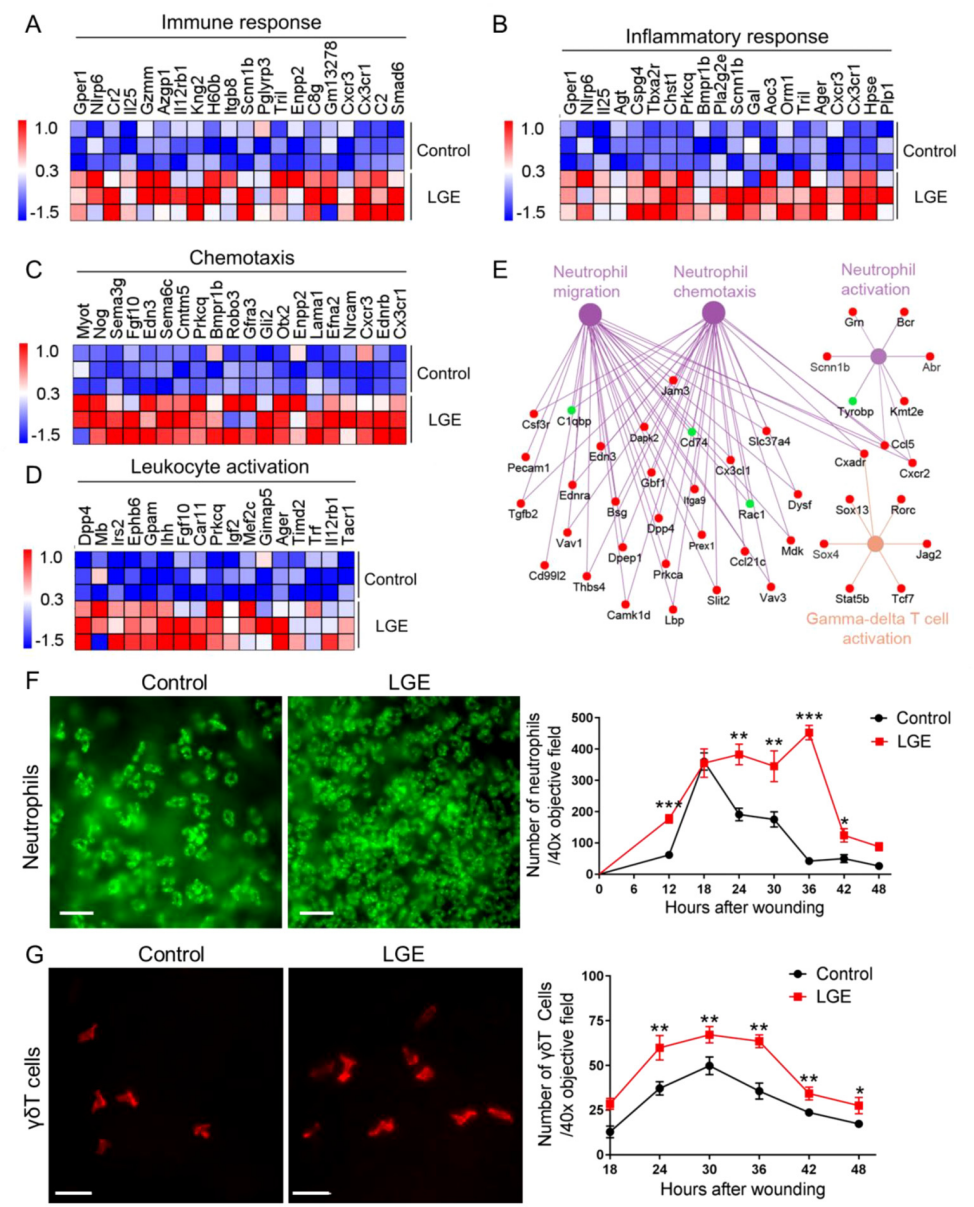
LGE enhances corneal inflammatory responses following epithelial abrasion. **(A–D)** Heatmaps showing differential expression of genes associated with immune response **(A)**, inflammatory response **(B)**, chemotaxis **(C)**, and leukocyte activation **(D)** in corneas of LGE‐treated mice compared with control mice. **(E)** Cytoscape network analysis of differentially expressed genes related to neutrophil migration, neutrophil chemotaxis, neutrophil activation, and γδ T cell activation in LGE‐treated corneas. Upregulated genes are shown in red, and downregulated genes are shown in green. **(F)** Representative immunofluorescence images (left) and quantification (right) of neutrophils infiltrating the wounded cornea at different time points post‐epithelial abrasion. Neutrophils were detected using an anti-Ly6G antibody conjugated with fluorescein isothiocyanate (FITC) and imaged under a 40× objective lens (scale bars = 25 μm). The number of neutrophils was counted in four non‐overlapping fields per sample (see **Supplementary Figure 2**). **(G)** Representative immunofluorescence images (left) and quantification (right) of γδ T cells infiltrating the cornea. γδ T cells were stained using an anti-GL3 antibody conjugated with phycoerythrin (PE) and visualized under a 40× objective lens (scale bars = 25 μm). Quantification was based on counts in four representative fields per sample (see **Supplementary Figure 2**). All data are presented as mean ± SD. Sample size: *n* = 3 independent experiments (4 mice per experiment in each group; **(A–E)**); *n* = 6 mice per group **(F, G)**. Statistical significance: *P < 0.05, **P < 0.01, and ***P < 0.001.

To systematically examine the impact of LGE‐induced aqueous‐deficient dry eye on corneal inflammation following epithelial injury, we conducted standardized transcriptomic profiling complemented by precise quantification of immune cell infiltration. RNA‐sequencing analyses, visualized through standardized heatmaps, consistently demonstrated widespread and significant upregulation of genes related to immune response, inflammatory response, chemotaxis, and leukocyte activation in corneas from LGE‐treated mice compared with controls (**Figures 3A–D**). Further, systematic network analyses using Cytoscape software reproducibly highlighted prominent activation of gene clusters specifically associated with neutrophil migration, chemotaxis, activation, and γδ T cell responses (**Figure 3E**).

To rigorously assess the temporal dynamics of immune cell infiltration, we performed standardized whole‐mount immunofluorescence staining. Quantitative analysis revealed that Ly6G^+^ neutrophil infiltration at the corneal wound margins was significantly elevated in LGE‐treated mice at multiple defined intervals (12, 24, 30, 36, and 42 hours post‐abrasion) compared with controls (**Figure 3F**). Similarly, standardized quantification of GL3‐labeled γδ T cells consistently showed significantly increased infiltration at 24, 30, 36, 42, and 48 hours following epithelial injury in the LGE group relative to controls (**Figure 3G**).

Collectively, these systematically obtained results clearly indicate that LGE‐induced aqueous‐deficient conditions significantly exacerbate corneal inflammation after epithelial injury, evidenced by robust increases in pro‐inflammatory gene expression and reproducibly enhanced infiltration of neutrophils and γδ T cells.

### Inhibition of the NF-κB and IL-6 signaling reduces corneal inflammation in LGE-treated mice

**Figure 4:**
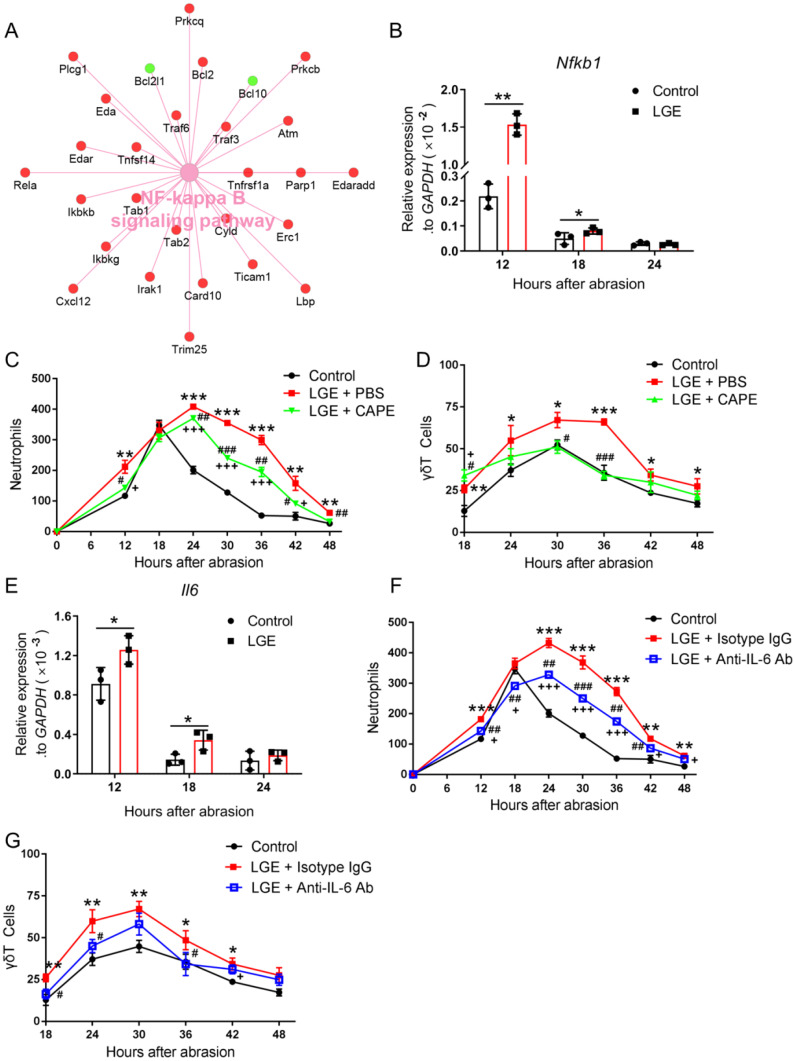
Inhibition of NF‐κB and IL‐6 signaling reduces corneal inflammation in LGE‐treated mice after epithelial abrasion. **(A)** Cytoscape network visualization of differentially expressed genes in the NF‐κB signaling pathway in corneas from LGE‐treated versus control mice. Upregulated genes are shown in red; downregulated genes in green. **(B)** Quantitative PCR analysis of *Nfkb1* mRNA levels in corneal tissue at 12, 18, and 24 hours after abrasion. **(C, D)** Quantification of neutrophils **(C)** and γδ T cells **(D)** in wounded corneas of control mice, LGE‐treated mice receiving PBS, and LGE‐treated mice treated with the NF-κB inhibitor CAPE. **(E)** Quantitative PCR analysis of *Il6* mRNA expression in corneal tissue at 12, 18, and 24 hours post‐abrasion. **(F, G)** Quantification of neutrophils **(F)** and γδ T cells **(G)** in corneas of control mice, LGE‐treated mice administered isotype IgG, and LGE‐treated mice administered anti‐IL‐6 neutralizing antibody. Infiltrating cells were counted in four non‐overlapping 40× objective fields per cornea. All data are shown as mean ± SD. Sample size: *n* = 3 independent experiments (4 mice per experiment in each group; **(A, B, E)**; *n* = 6 mice in each group **(C, D, F, G)**. Statistical significance: *P < 0.05, **P < 0.01, ***P < 0.001 for comparisons between LGE‐treated and control mice; ^#^ *P* < 0.05, ^##^ *P* < 0.01, ^###^ *P* < 0.001 for comparisons between CAPE or anti‐IL‐6‐treated LGE mice and vehicle‐ or isotype‐treated LGE mice; ^+^ *P* < 0.05, ^+++^ *P* < 0.001 for comparisons between CAPE or anti‐IL‐6‐treated LGE mice and control mice.

To rigorously investigate the role of inflammatory signaling pathways in LGE‐induced corneal inflammation, we first performed comprehensive transcriptomic profiling. Cytoscape‐based network analysis consistently revealed significant upregulation of numerous genes implicated in the NF‐κB signaling pathway in corneas of LGE‐treated mice compared with controls following epithelial abrasion (**Figure 4A**). Confirmatory quantitative PCR analysis reproducibly demonstrated significantly elevated expression of the key NF‐κB component, *Nfkb1*, at precisely 12 and 18 hours post‐injury in LGE‐treated mice (**Figure 4B**).

To systematically assess the functional relevance of NF‐κB pathway activation, we administered a standardized pharmacological inhibitor, caffeic acid phenethyl ester (CAPE), to LGE‐treated mice. Relative to PBS‐treated controls, CAPE administration reproducibly and significantly decreased neutrophil and γδ T cell infiltration into injured corneas at multiple standardized intervals post‐abrasion (**Figures 4C, D**).

Given that IL‐6 is a well‐documented downstream effector of NF‐κB implicated in corneal inflammation, we further examined *Il6* mRNA expression by standardized quantitative PCR. Corneal *Il6* levels were consistently and significantly elevated at 12 and 18 hours after injury in LGE‐treated mice relative to controls (**Figure 4E**).

To rigorously test IL‐6’s direct contribution to corneal inflammation, we topically applied a neutralizing anti‐IL‐6 antibody to the ocular surface of LGE‐treated mice. Standardized quantification revealed that IL‐6 blockade significantly reduced infiltration of both neutrophils and γδ T cells into wounded corneas compared to isotype IgG‐treated controls (**Figures 4F, G**).

Collectively, these precisely controlled and reproducible findings clearly demonstrate that pharmacological inhibition of NF‐κB or direct neutralization of IL‐6 effectively attenuates inflammatory cell infiltration in corneas under aqueous‐deficient dry eye conditions following epithelial injury, highlighting these pathways as critical inflammatory mediators.

### Inhibition of the NF-κB/IL-6 signaling axis promotes corneal wound healing in LGE-treated mice

**Figure 5:**
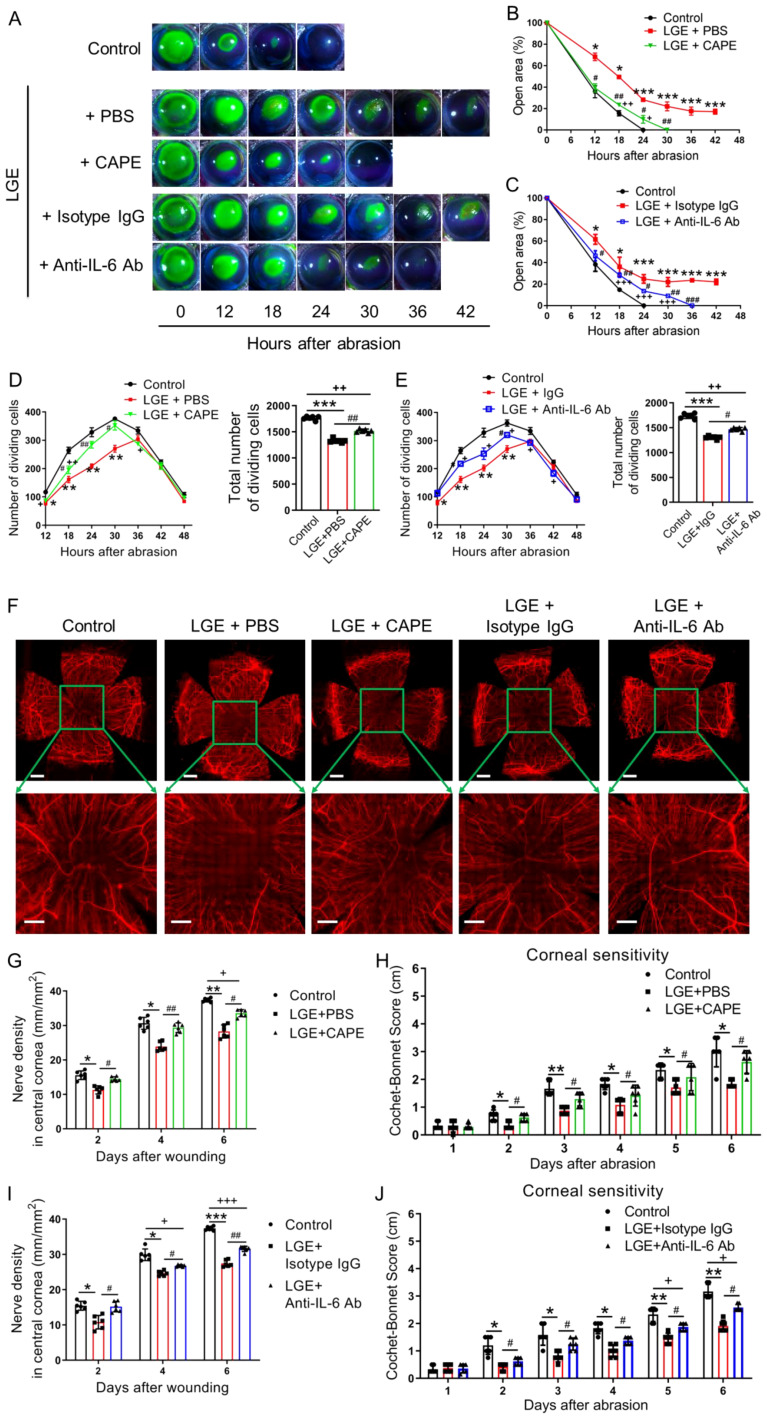
Effects of NF-κB or IL-6 inhibition on corneal re‐epithelialization and nerve regeneration in LGE-treated mice. **(A)** Representative fluorescein sodium staining images of corneal epithelial wounds at multiple time points (0–42 hours) after abrasion in control mice, and LGE‐treated mice administered PBS, CAPE, isotype IgG, or anti‐IL‐6 antibody. **(B, C)** Quantification of residual corneal wound area over time in each group of mice. **(B)** Comparison among control, LGE + PBS, and LGE + CAPE groups. **(C)** Comparison among control, LGE + isotype IgG, and LGE + anti‐IL‐6 antibody groups. **(D, E)** Quantification of dividing basal epithelial cells in each group of mice. **(D)** Comparison among control, LGE + PBS, and LGE + CAPE groups; **(E)** Comparison among control, LGE + isotype IgG, and LGE + anti‐IL‐6 antibody groups. **(F)** Immunofluorescence images of whole-mount corneal nerves at 6 days post‐injury using anti‐β‐III tubulin antibody conjugated with NL557. Upper panels show full corneal view (scale bars = 500 μm); lower panels show magnified central region (scale bars = 200 μm). **(G, H)** Quantification of corneal nerve fiber density **(G)** and mechanical sensitivity **(H)** in control, LGE + PBS, and LGE + CAPE groups. **(I, J)** Quantification of corneal nerve fiber density **(I)** and mechanical sensitivity **(J)** in control, LGE + isotype IgG, and LGE + anti‐IL‐6 antibody groups. All values are presented as mean ± SD. Sample size: *n* = 6 mice in each group **(B–E, G–J)**. Statistical significance: *P < 0.05, **P < 0.01, and ***P < 0.001 for LGE‐treated mice versus control mice; ^#^ *P* < 0.05, ^##^ *P* < 0.01, and ^###^ *P* < 0.001 for LGE + CAPE or anti‐IL‐6 antibody versus respective vehicle (PBS or IgG); ^+^ *P* < 0.05, ^++^ *P* < 0.01, and ^+++^ *P* < 0.001 for LGE + CAPE or anti‐IL‐6 antibody versus control.

To systematically investigate whether targeted inhibition of NF‐κB or IL‐6 signaling pathways could enhance corneal wound healing under aqueous‐deficient dry eye conditions, LGE‐treated mice were rigorously administered either the NF‐κB inhibitor caffeic acid phenethyl ester (CAPE) or a neutralizing anti‐IL‐6 antibody. Corneal re‐epithelialization was quantitatively monitored using standardized fluorescein staining protocols over a precisely controlled 42‐hour observation period following epithelial abrasion. Compared to vehicle‐treated LGE mice (PBS or isotype IgG), both CAPE and anti‐IL‐6 antibody treatments reproducibly and significantly accelerated corneal wound closure; however, healing rates consistently remained slower than in untreated control mice (**Figures 5A–C**).

To rigorously evaluate proliferative capacity during re‐epithelialization, mitotic basal epithelial cells and *Ki67* and *Pcna* gene expression were systematically quantified. Both CAPE and anti‐IL‐6 antibody treatments significantly increased the number of proliferating epithelial cells (**Figures 5D, E**) and the expression of *Ki67* and *Pcna* (**Supplementary Figure 4**) compared with their respective vehicle controls. Nevertheless, total mitotic cell counts in both treatment groups remained reproducibly lower than those observed in control mice.

The effects of pharmacological intervention on corneal nerve regeneration were assessed using standardized β‐III tubulin immunostaining 6 days post‐injury. Both CAPE and anti‐IL‐6 antibody treatments significantly enhanced nerve fiber density in the central cornea compared to vehicle‐treated LGE mice (**Figures 5F, G, I**). Similarly, standardized measurements of corneal mechanical sensitivity using the Cochet‐Bonnet esthesiometer demonstrated partial restoration of sensory function in both treatment groups relative to vehicle‐treated controls, although full recovery to untreated control levels was not achieved (**Figures 5H, J**).

Collectively, these systematically controlled and rigorously quantified findings clearly indicate that pharmacological inhibition of NF‐κB or IL‐6 signaling pathways partially restores epithelial proliferation, accelerates wound closure, and significantly improves nerve regeneration and sensory recovery under aqueous‐deficient dry eye conditions following corneal injury.

## Discussion

In this study, we investigated how aqueous‐deficient dry eye disease (DED) impairs corneal wound healing and identified the NF‐κB/IL‐6 signaling axis as a critical mediator of delayed epithelial and nerve regeneration. Using a validated mouse model of DED induced by extraorbital lacrimal gland excision (LGE), we demonstrated that sustained inflammation—characterized by neutrophil and γδ T‐cell infiltration—impedes epithelial repair and neural regeneration. Importantly, pharmacological inhibition of NF‐κB or IL‐6 significantly mitigated inflammation and partially restored wound healing, highlighting therapeutic targets to improve clinical outcomes in patients suffering from DED.

One key finding from our research is the identification of persistent inflammatory infiltration as a driver of impaired re‐epithelialization and nerve regeneration. Although neutrophils and γδ T cells are essential for early stages of corneal healing, their prolonged or excessive presence disrupts the finely tuned repair process, leading to chronic epithelial defects and impaired regeneration (9, 10). Transcriptomic profiling confirmed the marked upregulation of inflammatory mediators, notably NF‐κB and IL‐6, accompanied by suppression of metabolic genes necessary for efficient tissue repair. This inflammatory‐metabolic imbalance provides mechanistic insight into the compromised wound healing seen clinically in patients with severe DED (36–38).

Our results align with and extend recent evidence highlighting the inflammatory nature of DED and its detrimental impact on ocular surface homeostasis (36, 39, 40). Previous studies have reported the recruitment and activation of immune cells, including monocyte‐derived macrophages (41) and dendritic cells (42), as central events in DED‐associated inflammation. Consistent with these reports, our data confirm that neutrophil and γδ T cell infiltration are exacerbated under aqueous‐deficient conditions, underscoring the critical role of sustained inflammation in disrupting corneal regeneration. Additionally, earlier studies demonstrated NF‐κB pathway activation in experimental dry eye models, resulting in increased expression of inflammatory cytokines, such as IL‐1β and IL-18 (42–45). Our findings build upon this knowledge by directly linking the NF‐κB-dependent inflammatory response to impaired epithelial regeneration and nerve repair, thus filling a critical gap in our understanding of DED pathogenesis.

Mechanistically, our study provides robust evidence that the NF‐κB/IL‐6 signaling pathway serves as a central inflammatory amplifier in DED. NF‐κB activation, widely recognized as a critical regulator of ocular surface inflammation and epithelial integrity (46, 47), drives elevated IL‐6 expression (30, 48), thereby perpetuating the inflammatory milieu. Indeed, our pharmacological inhibition experiments using CAPE (an NF‐κB inhibitor) effectively reduced infiltration of inflammatory cells, validating NF‐κB’s role in sustaining inflammation following corneal injury. Moreover, neutralization of IL‐6 produced parallel anti‐inflammatory effects, significantly improving both epithelial proliferation and neural regeneration, thus confirming IL‐6 as a key downstream effector of NF‐κB‐mediated pathology (30, 48, 49). Although previous studies have implicated NF−κB or IL−6 in DED, our work is the first to simultaneously evaluate epithelial repair, nerve regeneration, immune cell infiltration, and functional sensory recovery in a dry eye model, thereby offering an integrative perspective on the healing process.

Our results demonstrate that delayed nerve regeneration coincides with inflammatory cell infiltration, and that blockade of either NF-κB or IL-6 not only alleviates inflammation but also promotes nerve regeneration and sensory recovery. This suggests that nerve repair is influenced by the inflammatory microenvironment. It is known that neurosensory deficits contribute significantly to chronic inflammatory states by impairing neuroimmune regulatory mechanisms (50). Additionally, our previous study indicated that sensory nerves expressing TRPV1 can suppress ocular inflammation through neuropeptide release (51), indicating that impaired nerve regeneration in DED may further disrupt inflammatory resolution. Together, these findings suggest a potential neuro‐immune interplay in DED. Future investigations should explore whether restoration of sensory nerve function can directly attenuate inflammation, thereby providing complementary therapeutic strategies alongside NF‐κB/IL‐6 inhibition.

Importantly, our findings offer significant translational relevance. Currently, therapeutic approaches to DED primarily focus on symptomatic relief through artificial tears or anti‐inflammatory eye drops, with limited regenerative capacity (36, 40). By specifically targeting the NF‐κB/IL‐6 axis, our study introduces a promising therapeutic strategy with the potential to restore ocular surface integrity and nerve function. This could greatly benefit patients undergoing ocular surgeries, such as refractive procedures, or those with chronic ocular surface disorders, where healing is often complicated by underlying dry eye pathology.

However, our study has limitations that should guide future research. First, although pharmacological inhibition of NF‐κB or IL‐6 demonstrated efficacy, the precise contributions of individual cell types (epithelial cells, nerves, immune cells) to the observed outcomes require clarification through cell‐specific conditional knockout approaches. Second, although we assessed proliferation through DAPI-stained nuclear division and qPCR analysis of *Ki67* and *Pcna*, these findings should be supplemented with immunohistochemical staining for Ki67 or PCNA protein to provide direct visual evidence. Third, we acknowledge that conclusions regarding cellular responses would be substantially strengthened by single-cell RNA sequencing of the entire cornea or bulk RNA sequencing of isolated corneal epithelial and stromal layers. Consequently, we plan to utilize single-cell RNA-seq in future work. Fourth, while mouse models offer valuable insights, direct extrapolation to human pathology demands caution due to anatomical and immunological differences. Finally, long‐term tissue remodeling and fibrosis, critical to understanding chronic dry eye pathology, were not assessed here and remain important areas for further investigation.

In conclusion, our study demonstrates that aqueous‐deficient dry eye significantly compromises corneal epithelial and nerve regeneration by sustaining inflammation mediated through the NF‐κB/IL‐6 signaling pathway. Targeted modulation of this axis markedly reduces inflammatory cell infiltration and partially restores regenerative capacities, highlighting its promise as a novel therapeutic strategy. This study not only enhances mechanistic understanding but also paves the way for translational interventions aimed at improving ocular surface health and vision‐related quality of life in patients suffering from dry eye disease.

## Data Availability

The datasets presented in this study can be found in online repositories. The names of the repository/repositories and accession number(s) can be found below: PRJNA1306019 (SRA).
