# Supplementary material for: NF-κB/IL-6 axis drives impaired corneal wound healing in aqueous-deficient dry eye
Source: Front Immunol. 2025 Oct 24;16:1684290. doi: 10.3389/fimmu.2025.1684290 (PMC12591958; doi:10.3389/fimmu.2025.1684290)
Supplement: Supplementary file 1 [file DataSheet1.docx]

**NF-κB/IL-6 Axis Drives Impaired Corneal Wound Healing in Aqueous-Deficient Dry Eye**

**Running Title: Targeting NF-κB/IL-6 in Dry Eye**

Cuipei Lin^1,2^, Jiaxin Wu^1,2^, Yuxi Jing^1,2^, Jingbin Xie^1,2^, Jiayan Xiang^1,2^, Qiwei Fan^1^, Jiangman Liu^1^, Jingheng Du^1^, Xiukui Tan^1^, Zhudan Zhuang^1^, Yunxia Xue^1^, Ting Fu^1^, Jun Liu^1,2*^, Zhijie Li^1,2*^

^1^International Ocular Surface Research Center, Institute of Ophthalmology, and Key Laboratory for Regenerative Medicine, Jinan University, Guangzhou, China;

^2^Department of Ophthalmology, The First Affiliated Hospital of Jinan University, Guangzhou, China;

^*^Corresponding author, email address: liujun_jnu@126.com, tzhijieli@jnu.edu.cn.


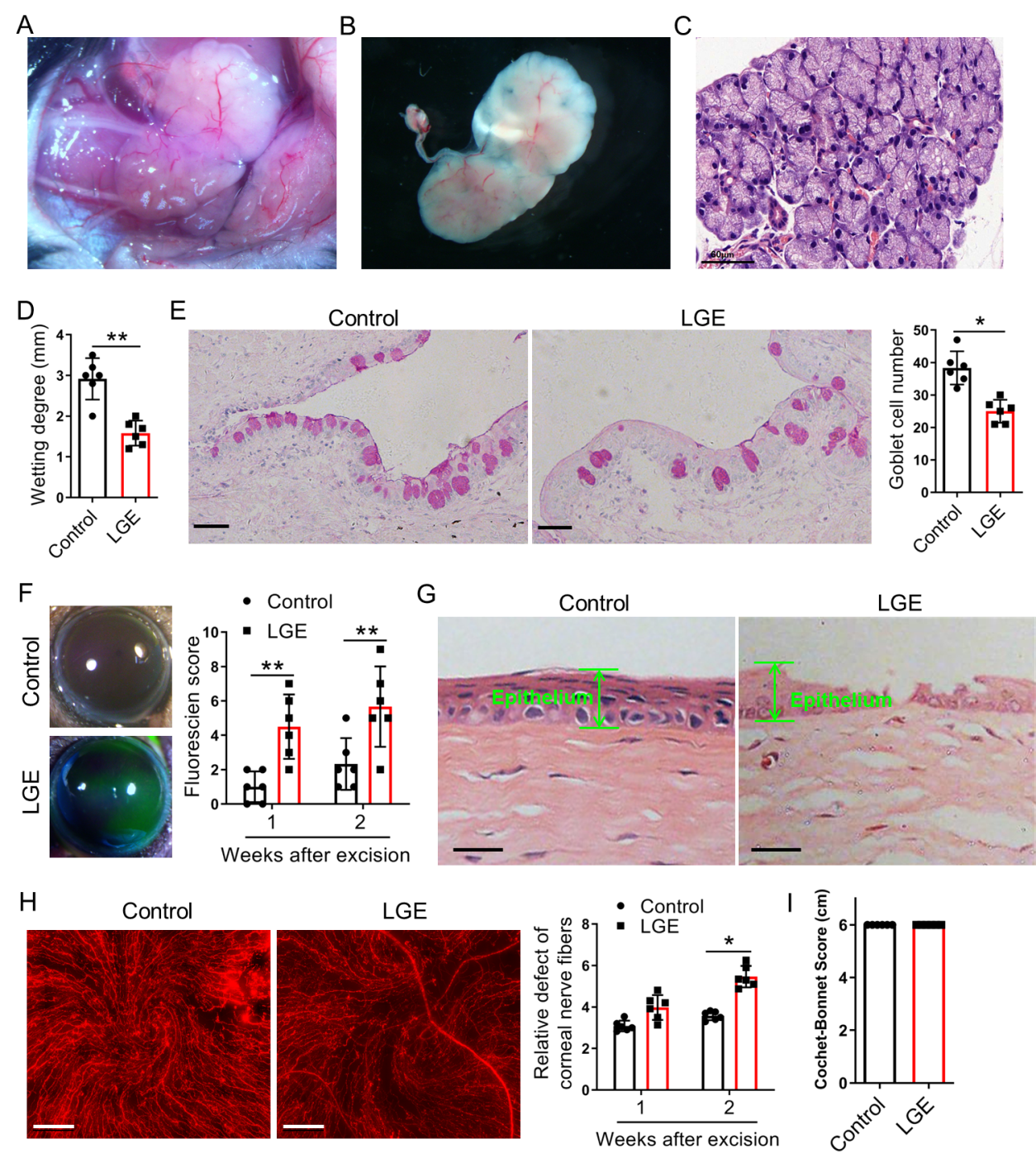


**Supplementary Figure 1. Establishment of an aqueous‑deficient dry eye (DED) mouse model by extraorbital lacrimal gland excision (LGE).**

(**A**) Intraoperative view showing the exposed extraorbital lacrimal gland between the eye and ear.

(**B**) Gross appearance of the lacrimal gland after excision.

(**C**) Representative hematoxylin and eosin (H&E) section of the excised gland (scale bar, 60 μm).

(**D**) Basal tear secretion measured with Schirmer tear test strips; LGE‑treated mice show reduced wetting length versus controls.

(**E**) H&E‑stained conjunctival sections from control and LGE‑treated mice illustrating reduced goblet cell counts in the LGE group (scale bars, 20 μm); right, quantitative comparison.

(**F**) Corneal fluorescein staining 2 weeks after LGE; left, representative images; right, comparison of staining scores between LGE‑treated and control mice.

(**G**) Representative H&E images of the central corneal epithelium 2 weeks post‑LGE (scale bars, 25 μm).

(**H**) Whole‑mount immunofluorescence of corneal nerve fibers at 2 weeks post‑LGE using anti–β‑III tubulin conjugated with NL557 (scale bars, 100 μm); right, quantification of relative nerve‑fiber loss in LGE‑treated versus control mice.

(**I**) Corneal mechanical sensitivity at 2 weeks post‑surgery measured with a Cochet-Bonnet esthesiometer.

All data are mean ± SD. Sample size: n = 6 mice per group (D, E, F, H, I). Statistical significance: **P* < 0.05; ***P* < 0.01.


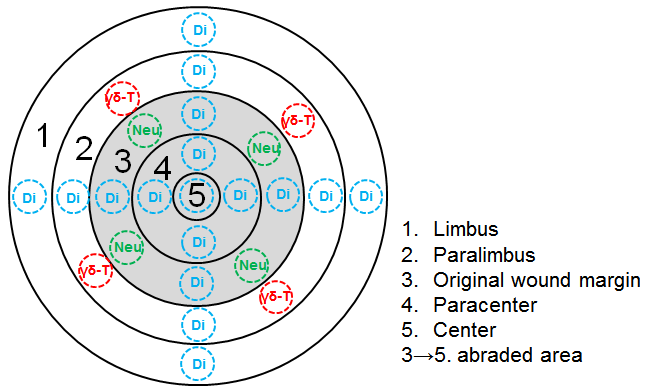


**Supplementary Figure 2. Schematic of field selection and quantification.** A whole‑mount cornea with intact limbus was divided into five concentric zones (1-5) from the limbus to the center; zones 3-5 delineate the abraded region.

(**A**) Epithelial proliferation: total number of mitotic epithelial cells across 17 40× fields (blue circles) spanning zones 1-5, reported per cornea.

(**B**) Neutrophil infiltration: mean neutrophil count from four 40× fields (green circles) positioned at the wound margin.

(**C**) γδ T‑cell infiltration: mean γδ T‑cell count from four 40× fields (red circles).

Abbreviations: Di, dividing epithelial cell; Neu, neutrophil.


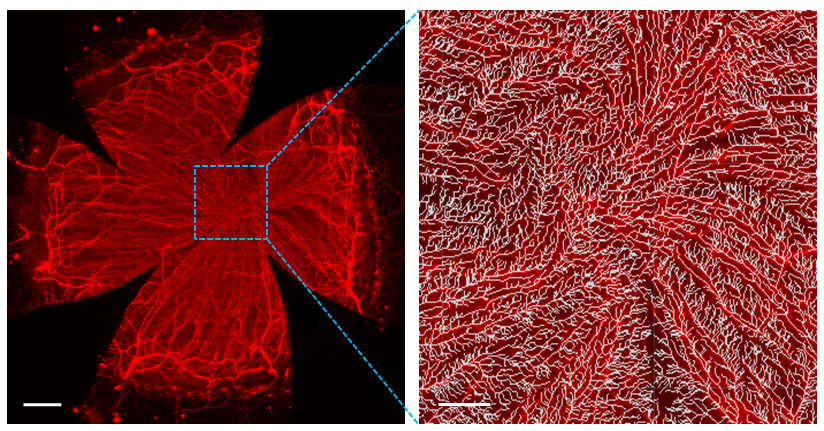


**Supplementary Figure 3. Workflow for quantifying central corneal nerve density.** Images of β‑III tubulin-labeled corneal nerves were imported into Imaris 6.2 (Bitplane AG, Zurich, Switzerland). A 1500 μm × 1500 μm central region of interest (ROI) was defined, the sum of nerve fiber lengths within the ROI was measured, and nerve density was expressed as total length per unit area (mm/mm²). Left: whole‑cornea view with ROI (scale bar, 500 μm). Right: magnified ROI (scale bar, 100 μm)


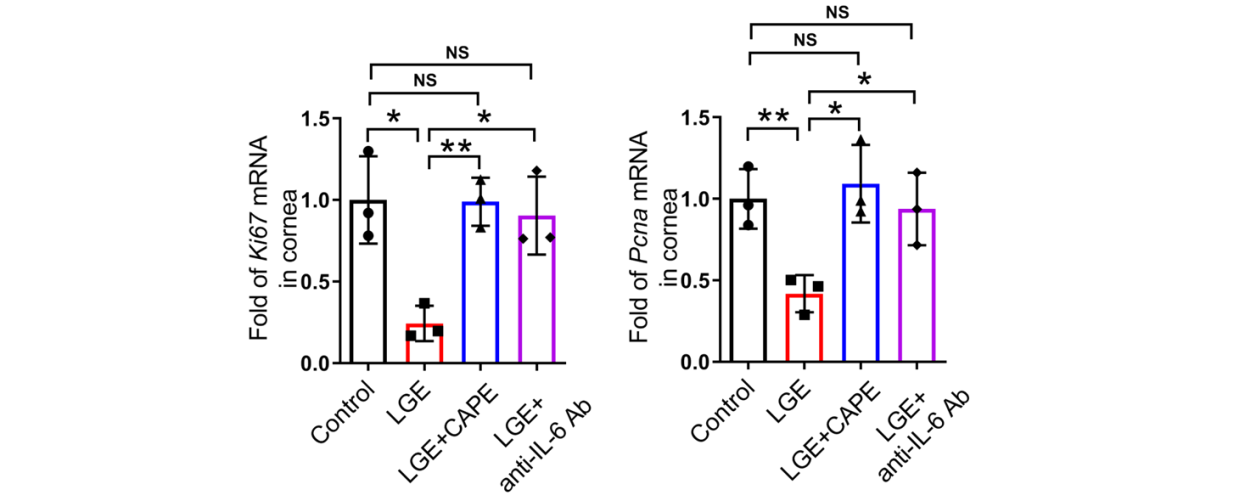


**Supplementary Figure 4. Quantitative PCR (qPCR) of *Ki67* and *Pcna* expression in corneal tissue from control, LGE, LGE+CAPE, and LGE+anti‑IL‑6 antibody groups.** qPCR reactions were run in technical triplicate (see Methods), and results are shown as mean±SD across 3 independent experiments (each experiment included 4 mice per group). Statistical significance: **P* < 0.05; ***P* < 0.01.
